# Intelligent fault tolerance control using long short-term memory for efficient system performance under fault conditions

**DOI:** 10.1038/s41598-025-99500-z

**Published:** 2025-05-09

**Authors:** Mostafa H. El-Mahdy, Abdelrahman O. Ali, O. H. Hassan, Eman M. El-Gendy, Mahmoud M. Saafan

**Affiliations:** 1 Mechatronics Department, Faculty of Engineering, Ahram Canadian University, Cairo, Egypt; 2 Mechanical Power Engineering Department, Faculty of Engineering, Mansoura University, Mansoura, Egypt; 3 Faculty of Engineering, Mansoura National University, Mansoura, Egypt; 4 Computers Engineering and Control Systems Department, Faculty of Engineering, Mansoura University, Mansoura, Egypt

**Keywords:** Passive fault tolerant controllers, Robust controller, Sensors faults, Assembler, And long short-term memory network, Electrical and electronic engineering, Mechanical engineering

## Abstract

Fault-Tolerant Control (FTC) is a crucial field within control systems engineering that focuses on designing systems capable of maintaining desired performance and stability even in the presence of faults. This study introduces a data-driven fault-tolerant control system that enhances the operation of control systems in the presence of faults. The system is designed on a single Long Short-Term Memory (LSTM), which replaces the units responsible for diagnosis and control reconfiguration. The LSTM-FTC system does not require diagnostic and process models, which is a significant advantage over traditional model-based methods. The factory I/O is interfaced with MATLAB through the implementation of the digital twin idea, which allows for the simulation and validation of the suggested approaches. These approaches are then applied to an assembler case study that included both faultless and multiple faulty sensors. The training process reaches 6553 iterations with Root Mean Square Error (RMSE) equal to $\:5\times\:{10}^{-3}$ at six minutes and 17 s. The results of the simulation demonstrate the effectiveness of the proposed approaches. The accuracy of the system outputs in the faultless and worst-case scenarios are 92.81% and 67.16% respectively.

## Introduction

Industry 4.0 focusses on the automation of traditional manufacturing processes and the optimization of the global production and supply network^1^. Also, with the huge growth in demand for product quality, system reliability, and plant availability, researchers and engineers have focused increasingly on developing techniques and systems that can withstand certain sorts of faults. The worldwide crisis intensifies the rivalry among businesses, making plant closures impractical due to the ensuing decrease in production and subsequent loss of market share^2^. Furthermore, malfunctioning equipment poses a significant threat to human survival in the industrial sector. On the other hand, modern technologies and challenging working conditions heighten the likelihood of system malfunctions, potentially leading to fatalities and equipment damage^3^. This is achieved using closed-loop control system components. These components are susceptible to malfunctions, particularly in sensors. Therefore, it is necessary to implement a Fault-Tolerant Control (FTC) system to effectively address various types of faults^4^.

FTC is a crucial field within control systems engineering that focuses on designing systems capable of maintaining desired performance and stability even in the presence of faults^5^. Faults can be unexpected events like sensor malfunctions, actuator failures, or process disturbances, all of which can jeopardize the system’s functionality and safety^6^. Modern control systems are becoming increasingly complex, incorporating more interconnected components and sensors. This complexity amplifies the likelihood of faults occurring, highlighting the need for robust strategies^7^.

Fault diagnosis has three essential tasks which are fault detection, fault identification, and fault isolation^8^. Fault detection is the procedure of ascertaining if a system is functioning in its usual state or if a malfunction has transpired. Fault identification is used to precisely determine the type of defect when it happens. Fault isolation is employed to precisely identify the malfunctioning system component(s). Nonlinear time-invariant dynamic systems may be categorized into two group of systems : continuous state & discrete state^9^. It is possible to classify discrete state systems as either event-driven or time-driven. Discrete Event System (DES) describes an event-driven system, whereas sampling describes a time-driven system. DES is a method of system modeling that uses rapidly altered signal levels to show the logical and sequential behavior of systems^10^.

**Fig. 1:**
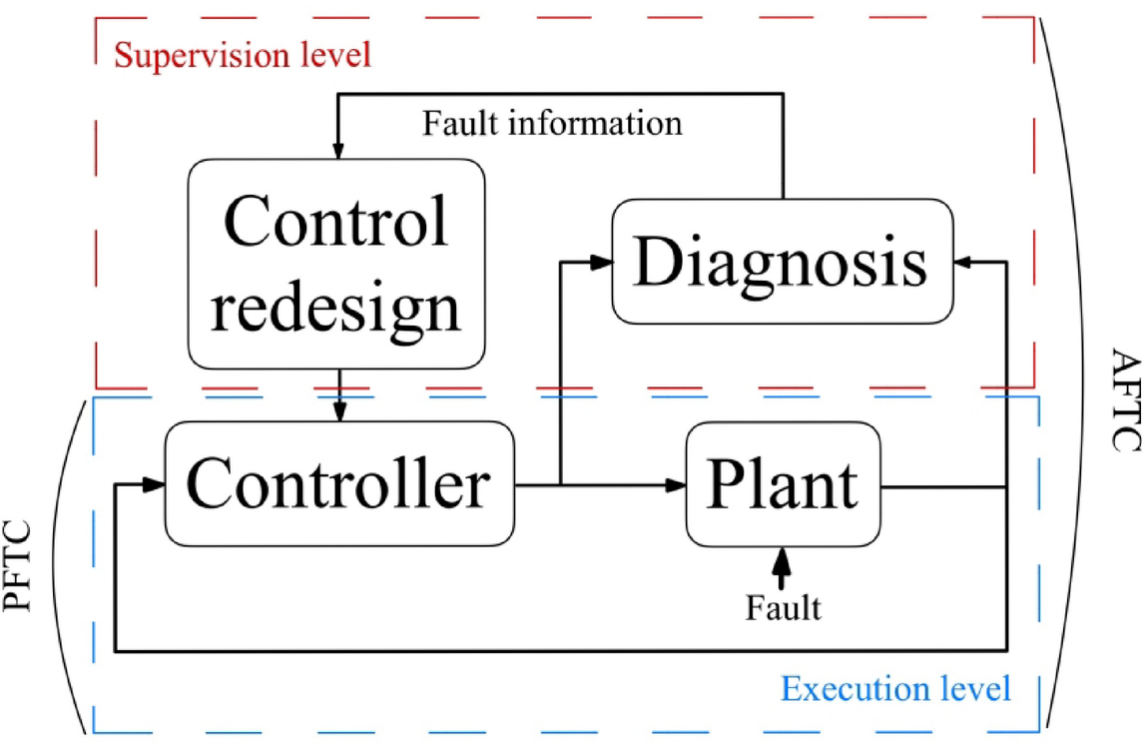
The architecture of FTC.

FTC techniques can be classified into two categories, as illustrated in Fig. 1^11^.

1. Passive FTC: these techniques rely on inherent system properties or pre-designed redundancy to tolerate faults without relying on real-time FDI^12^. Examples include voting systems for sensor redundancy or hardware redundancy for actuators.
2. Active FTC: these approaches actively detect and identify faults using FDI and then adapt the control strategy in real time to compensate for their effects^10^. This typically involves more complex design and computational resources.

Authors in^13^ developed a novel Fast Terminal Sliding Mode Surface (FTSMS) in order to address the singularity glitch and improve the convergence time of traditional Terminal Sliding Mode Control (TSMC). An innovative and robust FTCM has been created for robot manipulators. The purpose of this method is to achieve system stability, meet the desired performance criteria, and effectively counteract the negative impacts of disturbances, nonlinearities, or faults. The suggested FTCM exhibits notable characteristics, including rapid convergence rates, strong accuracy, superior tracking capabilities, substantial reduction of chattering behavior, and convergence within a defined period. The position tracking computer simulations were utilized to demonstrate the efficacy and viability of the proposed FTCM in comparison to alternative control algorithms. Le and Kang in^14^ presented a novel approach to achieve fault-tolerant control for a robot manipulator. The suggested method is based on synchronous sliding mode. As the synchronization errors decrease towards zero, the joint errors likewise decrease and begin to converge towards zero. Hence, the synchronization mechanism is naturally efficient for a fault-tolerant controller. A fault-tolerant controller utilizing a mix of synchronous sliding mode approach and estimator was suggested. Ultimately, fault tolerant control was applied to a robot manipulator with three degrees of freedom and then compared to the traditional sliding mode control. This comparison demonstrates the efficacy of the suggested active fault-tolerant control utilizing synchronous sliding mode approach. Ceglarek in^15^ developed A resilient FTC algorithm for robot manipulators, ensuring global fixed-time stability. This technique is formulated by combining a fixed-time second-order sliding mode observer with a fixed-time sliding mode control design strategy. The proposed system is designed to accurately predict the combined disturbance within a specific time frame. The suggested methodology is then implemented for the purpose of fault tolerant control of a PUMA560 robot and subsequently contrasted with various cutting-edge controllers. The simulation findings confirm the exceptional capacity of the suggested fault diagnosis observer and fault tolerant technique to estimate faults accurately and accommodate them effectively. The study in^16^ introduces a new Passive Fault Tolerant Control System (PFTCS) for regulating the Air Fuel Ratio (AFR) in Internal Combustion (IC) gasoline engines. The purpose of this system is to prevent the engine from shutting down. To regulate the AFR, a Proportional plus Integral (PI) controller with a substantial feedback gain is utilized. The effectiveness of the proposed PFTCS is evaluated by adding noise into the sensor readings. The simulation results in the MATLAB / Simulink environment show that the proposed PFTCS is resilient to sensor defects in both normal and noisy situations, without any deterioration in AFR. The stability study of the suggested controller is conducted by calculating the poles of the closed-loop system. The suggested controller’s dependability has been assessed by computing the probabilistic reliability function of the model.

The problem of FTC in Markovian Jump Systems (MJSs) that experience both nonlinearity and actuator failures concurrently^17^. This study utilizes the Radial Basis Function (RBF) Neural Network (NN) approach to accurately represent nonlinearity, for which no prior knowledge is provided. Subsequently, the adaptive backstepping method is utilized to develop a NN-based FTC solution, which effectively addresses the complex scenario under consideration. The suggested technique only utilizes two types of adaptive parameters to accomplish the goal of FTC. This results in a reduced computing load and expands its potential applications. Ultimately, the efficacy of the devised methodology is showcased on an actual system: a mobile robot with wheels and a manipulator. Authors in^18^ addressed the difficulty of designing model-based FTC algorithms for nonlinear systems. A novel approach for FTC in the presence of sensor errors is introduced, utilizing deep learning techniques. The proposed methodology eliminates the separate steps of fault identification and isolation, as well as controller design, by employing a single Recurrent Neural Networks (RNNs). The proposed approach utilizes an end-to-end deep FTC technique to address a mechatronic system consisting of a spherical inverted pendulum. The configuration of the pendulum is altered by reaction wheels, which are in turn controlled by electric motors. The modelling and experimental findings demonstrate that the suggested strategy is capable of effectively managing sudden failures that arise in link position/velocity sensors. While Elmahdy et al.^10^ propose a learning FTC of discrete event systems subjected to sensor faults based on RNN. The proposed network has both previous and current sensors signals as inputs and current actuators signals as outputs. The new data driven fault tolerant control replaces the diagnosis and control reconfiguration block function in the traditional one.

The FTC system’s block diagram is presented in^19^ based on the timed automata concept. The controller regulates flawed DES by manipulating controlled events, while the plant reacts by generating uncontrollable events. The reconfiguration model block’s job is to check the signal against the time model system. The time model system generates a new event, but the DES fails to reach it in time. The reconfiguration control law detects a defect and replaces the regular fault behavior controller with a timed controller based on the results of the time diagnostic. In the next step, the controller receives the timed control law and uses it to fix the malfunctioning plant. The suggested FTC design in^20^ integrates the durability and rapid convergence within a finite period of non-singular terminal synergetic control with the optimization characteristics of an interval type-2 fuzzy satin bowerbird algorithm. A Fault Detection and Diagnosis (FDD) module is constructed using an adaptive state-augmented extended Kalman filter. The suggested approach’s efficacy is evaluated by employing a simulated two-degree-of-freedom robotic manipulator exposed to several incorrect circumstances. The findings collected have verified the suggested approach’s fault tolerance capabilities and consistent performance.

Chang et al. presented a new adaptive fault-tolerant attitude control method for fixed-wing UAVs^21^. The system is based on the long short-term memory (LSTM) network and is designed to handle high dynamic disturbances and actuator defects. Firstly, the adaptive laws can adjust for the high dynamic disturbances. Additionally, the suggested Adaptive Fault-Tolerant Control (AFTC) approach may effectively address the actuator errors. Furthermore, the LSTM network is employed to mimic the unfamiliar and cumulative nonlinearities over time. Finally, numerical simulation results are shown to demonstrate the efficacy of the suggested strategy. A novel approach to active fault-tolerant control using an LSTM network is introduced in^22^, which can withstand numerous faults in sensors. Two networks are utilized: the first network serves as a diagnostic tool for DES, capable of detecting faults in sensors. The second network functions as a reverse model of these systems, determining the appropriate control actions to be taken when sensors are not operating as intended. Park et al.^23^ proposed a combined learning technique for fault detection and identification in time series data. Their approach integrates an autoencoder for fault detection and an LSTM network for fault classification. Through a simulation study on an industrial process, their method demonstrated a 16.9% improvement in accuracy compared to deep Convolutional Neural Networks (CNNs). Abdelhameed and Darabi in^24^ proposed an innovative method utilizing RNNs for AFTC in ASMS with faulty sensors. Their approach employs two RNNs: the first for fault diagnosis and detection, and the second for control reconfiguration to determine appropriate control actions under faulty conditions. Experimental testing on a clamp and operation hydraulic circuit demonstrated the effectiveness of the method, achieving a Sum of Squared Errors (SSE) of 10^− 4^ with a total training time of approximately 6 + 2 min. An advanced AFTC method for AMS using LSTM networks for both fault diagnosis and control reconfiguration is presented in^25^. Unlike traditional methods requiring controller remodeling, the proposed approach retrains LSTM networks with updated sequence data. Compared to^24^, which used RNNs, this method enhances fault handling, enabling AMS operation despite multiple sensor failures. A digital twin framework integrates the Factory I/O simulator with MATLAB for validation, achieving a Root Mean Square Error (RMSE)) of 10^− 3^, 66.6% fault coverage, and a training time of 13 + 8 min.

The literature review highlights various FTC approaches, including sliding mode control, neural networks, and deep learning, tailored to different applications such as robot manipulators, UAVs, and automated manufacturing systems. Recent advancements integrate learning-based fault detection, reducing reliance on explicit fault diagnosis and reconfiguration. While deep learning methods enhance adaptability, they come with higher computational demands. Digital twins and simulation-based validation (e.g., Factory I/O, MATLAB) are increasingly used, though real-world experimental validation remains limited. Table 1 provides a comprehensive summary of the research on FTC.

A comparison of the relevant research clearly demonstrates that all of the structures proposed in previous studies have effectively tackled the problem of algorithm efficiency. However, traditional FTC in DES with sensor defects has some challenges:

1. Complexity: Traditional FTC systems for DES often involve multiple components, such as separate diagnostic and control reconfiguration units. This can make them complex to design, implement, and maintain.
2. Limited adaptability: Traditional FTC systems may not be able to adapt well to different types of sensor defects or changes in the system dynamics.
3. Computational cost: Running multiple separate units for diagnostics and control can be computationally expensive, especially for resource-constrained systems.

**Table 1:** Comprehensive summary of the research on FTC.

References	Methodology	Application	Key findings
^13^	Fast Terminal Sliding Mode Surface (FTSMS)	Robot manipulators	Improved convergence time, reduced chattering, superior tracking, stability under disturbances
^14^	Synchronous Sliding Mode Control	Robot manipulator (3DOF)	Effective fault-tolerant control through synchronization, compared with traditional SMC
^15^	Fixed-time Second-Order Sliding Mode Observer	PUMA560 robot	Ensures global fixed-time stability, accurately predicts disturbances, superior fault accommodation
^16^	Passive Fault-Tolerant Control (PFTC)	Internal Combustion engine AFR regulation	Resilient to sensor noise and defects, maintains stable AFR without deterioration
^17^	Radial Basis Function Neural Adaptive Backstepping	Markovian Jump Systems (MJS)	Handles nonlinearities and actuator failures with reduced computing load, validated on a mobile robot
^18^	Deep Learning-based FTC	Spherical inverted pendulum	End-to-end Recurrent Neural Network (RNN)-based FTC, manages sensor faults effectively
^10^	Learning-based FTC using RNN	Discrete Event Systems (DES)	Data-driven approach replaces traditional diagnosis and reconfiguration blocks
^19^	Timed Automata-Based FTC	Faulty DES	Time-based reconfiguration for fault detection and correction
^20^	Non-singular Terminal Synergetic Control + Interval Type-2 Fuzzy Algorithm	2-DOF robotic manipulator	Combines rapid convergence with optimization, verified fault tolerance
^21^	Adaptive FTC with LSTM	Fixed-wing UAVs	Adapts to high dynamic disturbances and actuator defects using LSTM networks
^22^	Active FTC with LSTM	Discrete Event Systems (DES)	Dual LSTM networks for fault diagnosis and control action determination
^23^	Autoencoder + LSTM	Industrial Process	16.9% accuracy improvement over deep CNN in fault detection and classification
^24^	RNN-based AFTC	Automated Sequential Manufacturing Systems (ASMS)	SSE of 10^(-4), training time: 6 + 2” " min, dual RNNs for diagnosis and control reconfiguration
^25^	LSTM-based AFTC	Automated Manufacturing Systems (AMS)	RMSE of $\:{10}^{-3},66.6\text{\%}$ fault coverage, training time: $\:13+8\text{}\text{m}\text{i}\text{n}$, handles multiple sensor failures

The contribution of this work can be summarized as follows:

- Presents a novel approach for FTC of DES in the presence of sensor defects.
- The proposed method utilizes a single LSTM model as a robust controller to replace both the diagnostic and control reconfiguration units.
- This controller is capable of properly controlling the AMS in the event of undiagnosable sensor failures based on time series.
- The sensors’ signals and their time sequence states are the LSTM’s inputs, whereas the LSTM’s outputs are the actuators’ signals, and their time series states.
- The Factory I/O simulation software is linked to MATLAB through the concept of a digital twin, which enables precise simulation and validation of the proposed solution.
- The study presents both flawless and flawed possibilities.

The organization of this paper is as follows: The “Architecture of the proposed system and design of deep learning-based FTC” section offers a visual representation of the suggested methodology. “Case study: assembler” section presents a case study on automated material handling to illustrate the application of the recommended method. The implementation of the assembler case study simulation is conducted in “Results and discussions” section. A detailed explanation of the discoveries and additional examination is presented in “Conclusion” section.

## Architecture of the proposed system and design of deep learning-based FTC

### Preliminaries

**Fig. 2:**
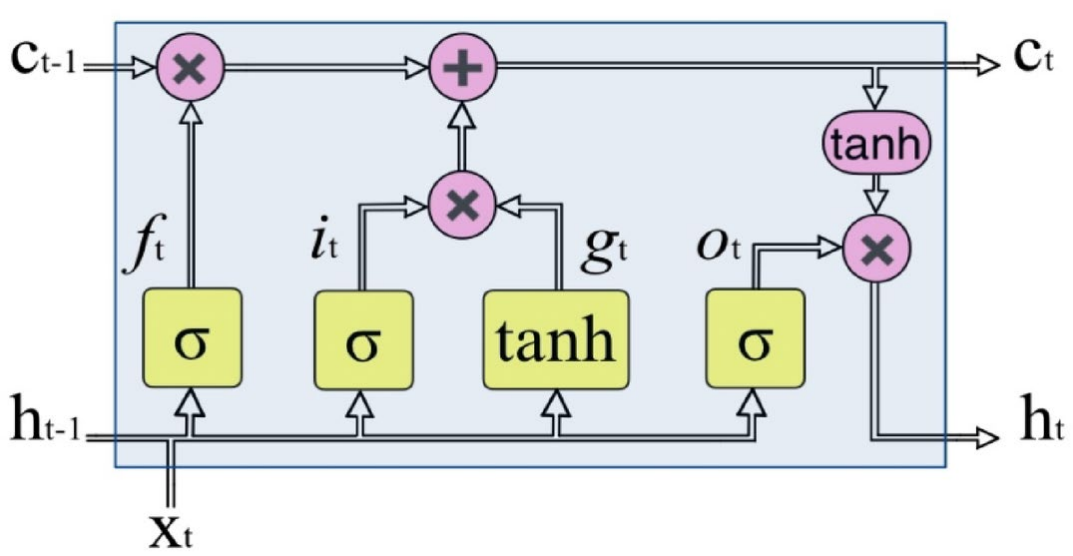
The data flow of the LSTM at time step t.

The LSTM is a specific type of RNN that excels at capturing and understanding long-term dependencies in data. It addresses the major drawbacks of RNNs, including the issues of gradient explosion along with gradient vanishing^26^. Figure 2 illustrates the data flow of the LSTM at a certain time step, t. This data flow is a crucial component of LSTM networks. This network consists of four layers of neural networks, namely the input-gate$\:\left({i}_{t}\right)$, the forget-gate$\:\left({f}_{t}\right)$, the output-gate$\:\left({o}_{t}\right)$, and the layer-input$\:\left({g}_{t}\right)$^27^. The $\:{i}_{t}$, $\:{f}_{t}$, and $\:{o}_{t}$ employ sigmoid $\:\left(\sigma\:\right)$ activation functions to respectively update, erase or reset, and incorporate the state of the cell into the output state. Conversely, the $\:{g}_{t}$ utilizes hyperbolic tangent $\:\left(tanh\right)$ activation functions to introduce information into the state of the cell. The mathematical equations representing these four levels are given by Eqs. (1–4)^28^.

1 $$\:{i}_{t}=\sigma\:\left({W}_{i}{x}_{t}+{R}_{i}{h}_{t-1}+{b}_{i}\right)\:$$

2 $$\:{f}_{t}=\sigma\:\left({W}_{f}{x}_{t}+{R}_{f}{h}_{t-1}+{b}_{f}\right)$$

3 $$\:{o}_{t}=\sigma\:\left({W}_{o}{x}_{t}+{R}_{o}{h}_{t-1}+{b}_{o}\right)\:$$

4 $$\:{g}_{t}={tanh}\left({W}_{g}{x}_{t}+{R}_{g}{h}_{t-1}+{b}_{g}\right)$$

Where:

- $\:t$ indicates the time step.
- $\:{W}_{i}$, $\:{W}_{f}$, $\:{W}_{o}$, and $\:{W}_{g}$ indicate the weights of the $\:{i}_{t}$, $\:{f}_{t}$, $\:{o}_{t}$, and $\:{g}_{t}$, in that order.
- $\:x$ indicates the input state.
- $\:{R}_{i}$, $\:{R}_{f}$, $\:{R}_{o}$, and $\:{R}_{g}$ indicate the recurrent weights of the $\:{i}_{t}$, $\:{f}_{t}$, $\:{o}_{t}$, and $\:{g}_{t}$, in that order.
- $\:h$ indicates the hidden state.
- $\:{b}_{i}$, $\:{b}_{f}$, $\:{b}_{o}$, and $\:{b}_{g}$ indicate the biases of the $\:{i}_{t}$, $\:{f}_{t}$, $\:{o}_{t}$, and $\:{g}_{t}$, in that order.

The output state is identified according to the equation shown as follows:

5 $$\:{h}_{t}={o}_{t}\:*\:{tanh}\left({c}_{t}\right)$$

Where $\:{c}_{t}$ indicates the cell state, which is identified according to the equation shown as follows:

6 $$\:{c}_{t}={f}_{t}*{c}_{t-1}+{i}_{t}*{g}_{t}$$

### Configuration of the fault tolerant control system

**Fig. 3:**
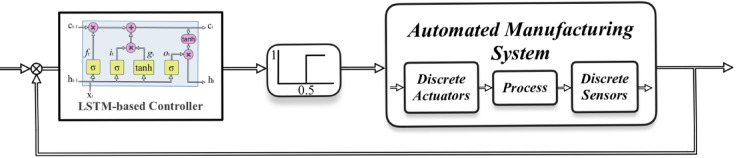
The block diagram of FTC using the LSTM.

Figure 3. depicts the block diagram of the fault tolerant control system employing a solitary LSTM. Automated Manufacturing Systems (AMS) is an acronym that denotes a dynamic system comprising many manufacturing processes^29^. This system is prone to sensor malfunctions. Based on the characteristics of AMS outlined in the previous chapter, LSTM has been selected as a reliable controller. The step function is employed to modify the signals of actuators. The inputs of the LSTM consist of the signals from the sensors and their corresponding time series states. On the other hand, the outputs of the network are the signals from the actuators and their respective time series states.

### Training of LSTM

**Fig. 4:**
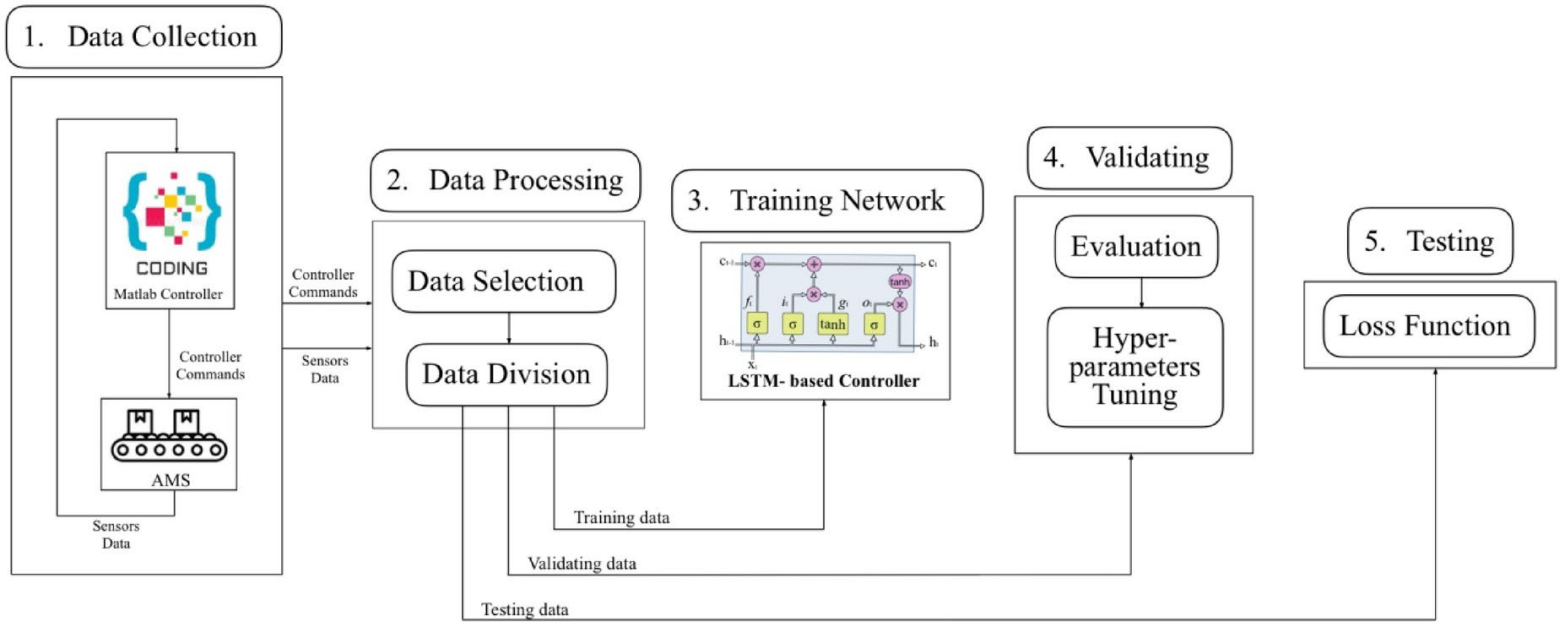
Training Approach for LSTM-based Controller.

Figure 4. demonstrates the training approach, which has five unique stages: Data Collection, Data Processing, Training Network, Validating phase, and Testing phase^30^. These stages are the essential processes for creating the intended network model.

#### Data collection stage

Supervised learning is conducted with a thorough understanding of the behavior of the AMS. The training data set is obtained by running the healthy AMS with the if-rule-based programming code, resulting in the extraction of both the input and output data sets. These training sets consist of two separate matrices: the input training matrix [$\:{X}_{{LSTM}_{i,j}}$] wherein $\:i=\:\left[1:\:q\right]$, $\:j=\:\left[1:t\:\right]\:$wherein $\:t$ is the overall count of time series provided by the AMS function, and the output training matrix [$\:{Y}_{{LSTM}_{i,j}}$] wherein $\:i=\:\left[1:\:p\right]$,wherein $\:j=\:\left[1:t\:\right]$. The format of the training matrices for input and output is as follows:

7 $$\left[ {X_{{LSTM_{{i,j}} }} } \right] = \left[ {\begin{array}{*{20}l} {u_{{H_{{1,1}} }} } \hfill & {u_{{H_{{1,2}} }} } \hfill & \cdots \hfill & {u_{{H_{{1,t}} }} } \hfill \\ {u_{{H_{{2,1}} }} } \hfill & {u_{{H_{{2,2}} }} } \hfill & \cdots \hfill & {u_{{H_{{2,t}} }} } \hfill \\ \vdots \hfill & \vdots \hfill & \ddots \hfill & \vdots \hfill \\ {u_{{H_{{q,1}} }} } \hfill & {u_{{H_{{q,2}} }} } \hfill & \cdots \hfill & {u_{{H_{{q,t}} }} } \hfill \\ \end{array} } \right]$$

8 $$\left[ {Y_{{LSTM_{{i,j}} }} } \right] = \left[ {\begin{array}{*{20}l} {y_{{A_{{1,1}} }} } \hfill & {y_{{A_{{1,2}} }} } \hfill & \cdots \hfill & {y_{{A_{{1,t}} }} } \hfill \\ {y_{{A_{{2,1}} }} } \hfill & {y_{{A_{{2,2}} }} } \hfill & \cdots \hfill & {y_{{A_{{2,t}} }} } \hfill \\ \vdots \hfill & \vdots \hfill & \ddots \hfill & \vdots \hfill \\ {y_{{A_{{p,1}} }} } \hfill & {y_{{A_{{p,2}} }} } \hfill & \cdots \hfill & {y_{{A_{{p,t}} }} } \hfill \\ \end{array} } \right]$$

#### Data processing stage

The data processing stage has two separate phases: selection and division of data^31^. After completing the data collection stage, the initial step is to meticulously choose the suitable training data that includes the whole healthy process of AMS. The objective is to incorporate the essential system parameters into the training data in a highly effective manner. The dataset is subsequently divided into subsets for training, testing, and validation in preparation for eventual utilization. During the training phase, precisely 80% of the entire data set is used. While the remaining data is utilized for the purpose of testing and validation, Each stage has been assigned a specific percentage of the dataset, which is 10%.

#### Training stage

The network’s biases and weights are optimized using Adam, an algorithm that adapts moment estimation, in order to minimize the function of losses^32^. Adam utilizes adjustable learning rates for different parameters, which have the ability to automatically adapt to the optimal loss function^33^. The system computes and retains the mean gradients and their squared magnitudes for every parameter, treating each element individually. Adam utilizes these average numbers to adjust the network configuration in the subsequent manner^34^:

9 $$\:{\theta\:}_{i+1}={\theta\:}_{i}-\frac{\alpha\:{m}_{i}}{\sqrt{{v}_{i}}+\epsilon\:}$$

Where $\:\theta\:$ represents the parameter vector, $\:i$ represents the iteration number, $\:{v}_{i}$ and $\:{m}_{i}$ represent the squared parameter gradients and the parameter gradients, respectively, $\:\epsilon\:$ represents the epsilon, and $\:\alpha\:$ represents the learning rate. The $\:{v}_{i}$ and $\:{m}_{i}$ are computed according to Eqs. 10 and 11, respectively.

10 $$\:{v}_{i}={\beta\:}_{v}{v}_{i-1}+\left(1-{\beta\:}_{v}\right){[\nabla\:E\left({\theta\:}_{i}\right)]}^{2}$$

11 $$\:{m}_{i}={\beta\:}_{m}{m}_{i-1}+\left(1-{\beta\:}_{m}\right)\nabla\:E\left({\theta\:}_{i}\right)$$

Where $\:{\beta\:}_{v}$ and $\:{\beta\:}_{m}$ represent the squared decay factor and the decay factor, respectively, and $\:\nabla\:E\left(\theta\:\right)$ represents the gradient of the loss function.

The training stage begins by assigning initial random values to the weights, which are sampled from a Gaussian distribution with a median of zero and the standard deviation of 0.01. The initial bias value is set to zero.

#### Validation stage

The models trained in stage 3 are evaluated for accuracy utilizing the validation data obtained in stage 2. LSTM models are optimized by modifying the network’s hyperparameters, which encompass the initial $\:\alpha\:$, $\:{\beta\:}_{m}$, $\:{\beta\:}_{v}$, batch size, and number of iterations, in accordance with the validation results^35^.

#### Testing stage

In this stage, the network models that have been optimized using the most effective hyper-parameters are evaluated utilizing the data obtained in stage 2. Subsequently, the evaluation of these enhanced network models is conducted.

### Design algorithm for FTC

Algorithm 1 provides a clear and precise description of the procedural steps involved in developing the LSTM-based FTC system. During the data gathering phase, a dataset is first extracted using the typical control program free of errors system. The LSTM training data sets are expressed as matrices, as seen in Eqs. (5–8), correspondingly. Subsequently, the LSTM structure is constructed. During the training process, the LSTM is trained offline using the Adam solver to accurately adjust weights and biases. Finally, the trained LSTM model is employed in a practical situation to analyse the data collected by the sensor. Thus, the network implements the feed-forward calculation to find out the anticipated output signals. The signals generated by the trained network are modified by the step block before their transmission to the actuators^36^.

**Algorithm 1:**
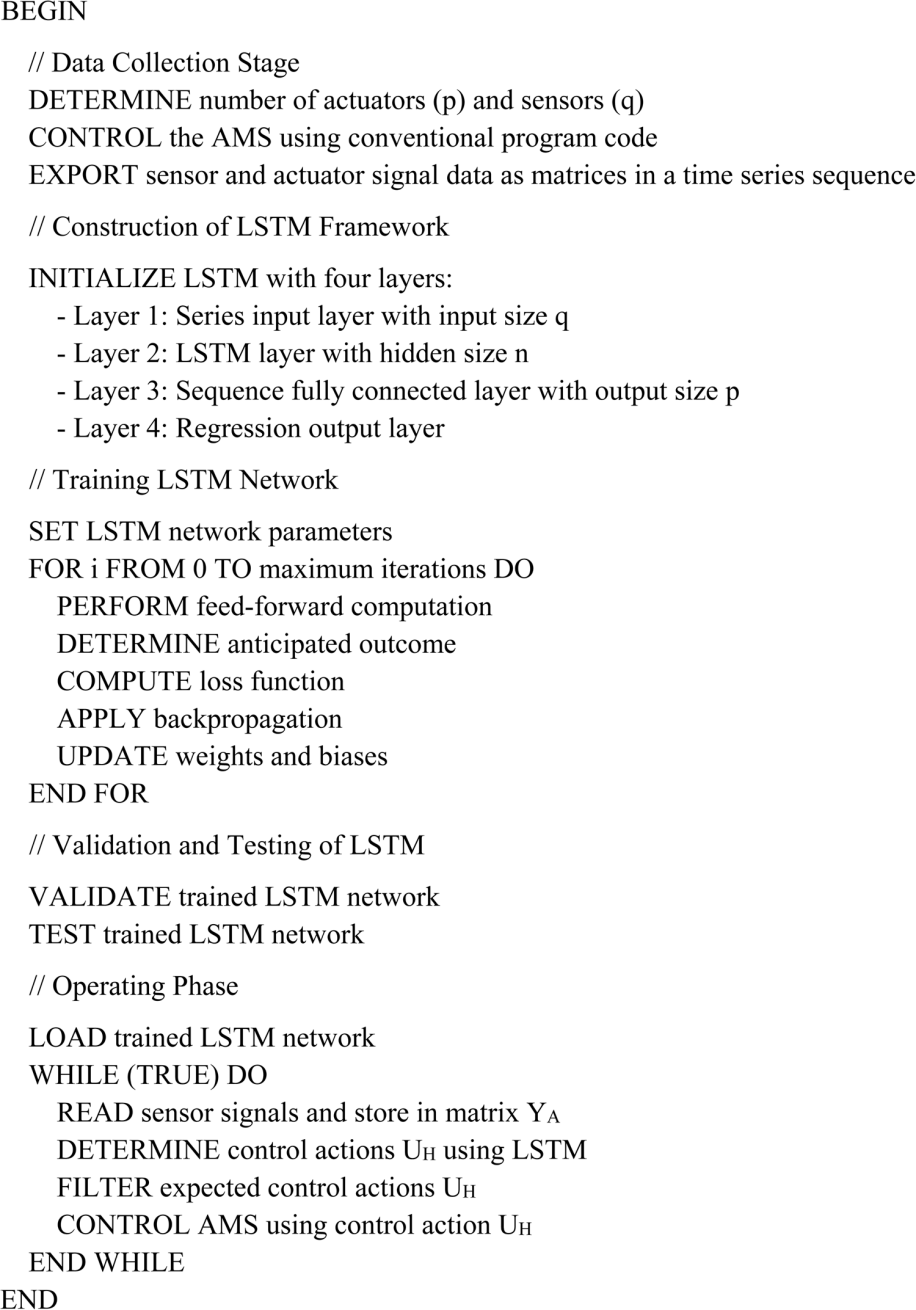
Design procedure of the FTC.

## Case study: assembler

The applied case study is an assembly line, a manufacturing process, sequentially adds components to a semi-finished assembly as it moves from workstation to workstation, ultimately producing the final assembly. By transferring the semi-completed assembly from one workstation to another and mechanically transporting the components to the assembly work, it is possible to assemble a finished product more quickly and with less labor than if employees were to convey the components to a stationary piece for assembly. The assembly line is a typical method of constructing complicated products, including vehicles, appliances, and electronics^37^.

**Fig. 5:**
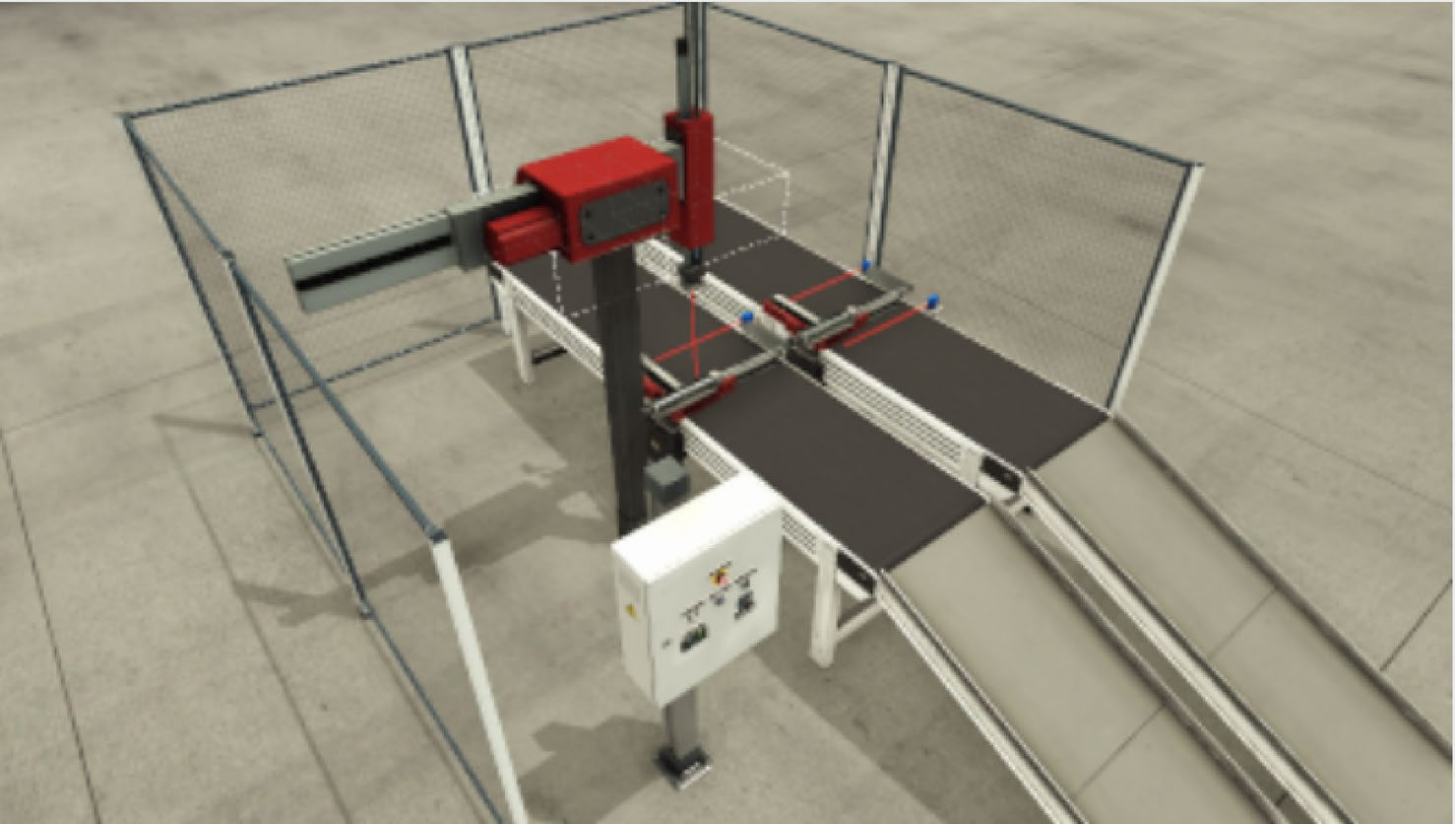
The factory IO assembler scene.

The assembly line used in this paper is shown in Fig. 5, which consists of a two-axis pick and place robotic arm with a vacuum gripper, two conveyor belts, and three single-acting cylinders, which make the actuation of the systems. The two-axis pick and place robotic arm is used to transfer lid components to base components, while the two conveyer belts are used to carry the lid and base components on the workstation. Two single-acting cylinders are used to clamp the base and lid objects while the other single-acting cylinder is used to lift the clampers to let the assembly move out. Two sensors for two-axis moving detection and one proximity sensor to detect the lid at the vacuum gripper. Two proximity sensors detect the lid and base at their places, and the third proximity sensor detects the assembly is leaving the workstation. Two normal open sensors for the clamped cylinder to detect the clamped components. One normal closed sensor to detect lift clamper single-acting cylinder at its initial position. Start switching to initialization of the system.

### Simulation software setup

**Fig. 6:**
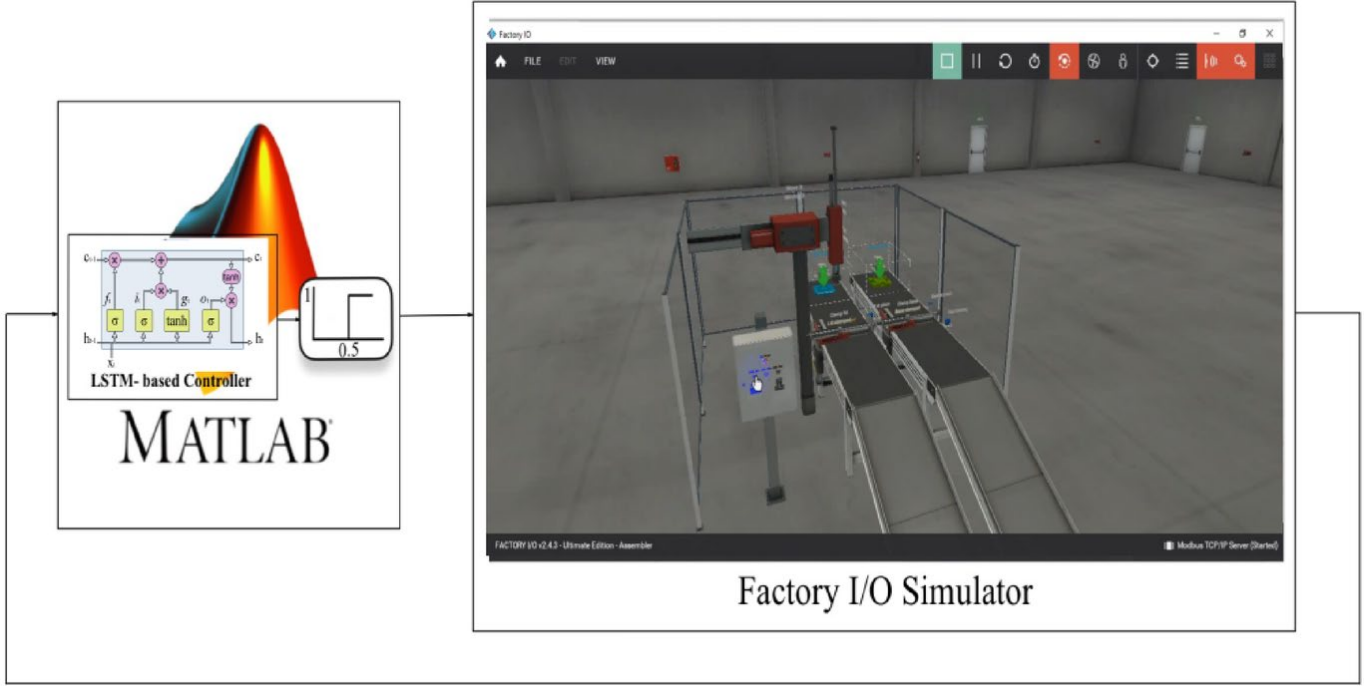
Simulation software setup.

The proposed AFTC is simulated and validated using the digital twin concept before it is implemented in a physical manufacturing system. A digital representation of the physical environment is referred to as a “digital twin”^38^. Modbus TCP/IP is the communication protocol that is utilized to interface the Factory I/O with MATLAB. The virtual system that was implemented in Factory I/O is the assembler case study. The Modbus server is eventually selected for MATLAB interaction once the factory I/O driver is enabled. An interface between MATLAB and the Factory I/O is utilized to implement the proposed AFTC, as depicted in Fig. 6. The MATLAB program code contains the LSTM with a unit step function to saturate the output signal of the trained LSTM, which acts as the robust controller.

### Construction of the proposed robust controller

The sensors involved in the assembler case study are two moving detection sensors, four proximity sensors, two normally open switches, one normally closed switch, and a start switch. Therefore, the number of inputs is equal to ten, while the actuators of the assembler case study are two axes pick and place robot arms, a suction gripper, two conveyor belts, and three single-acting cylinders. So, the number of actuators is equal to eight. As a result, the architecture of the LSTM consists of four layers, which are the sequence input layer with ten nodes, the LSTM hidden layer with 100 nodes, and the sequence fully connected layer with 8 nodes of regression output.

### LSTM training

**Fig. 7:**
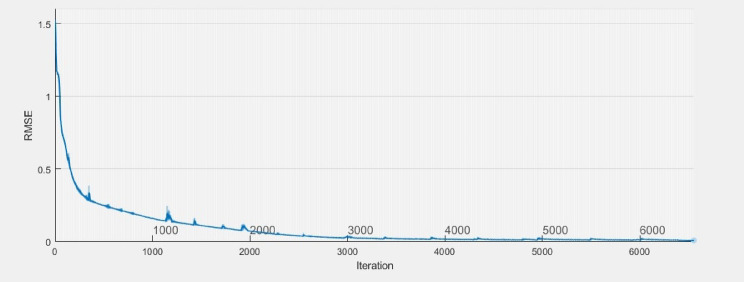
The training progress of the LSTM.

The learning process completes 6553 iterations in six minutes and 17 s. The RMSE is $\:5\times\:{10}^{-3}$ as shown in Fig. 7.

### Simulation

Extensive simulations were carried out as shifted binary code to show the performance of the robust controller based on the LSTM. In this section, four simulations are presented as follows:

Simulation one illustrates the functioning of the LSTM based controller for the faultless scenario.

Simulation two illustrates the functioning of the LSTM based controller when creating a fault in each sensor alone.

Simulation three illustrates the functioning of the LSTM based controller when creating faults in the nine sensors to show the performance of the proposed approach in the worst scenario.

## Results and discussions

In this section, the outcomes of the three simulations are presented. These simulations have been utilized to evaluate the performance of the proposed method. In contrast to an alternative method, the benefits of the proposed AFTC scheme are investigated as well in the discussion section.

### Results of the three simulations

**Fig. 8:**
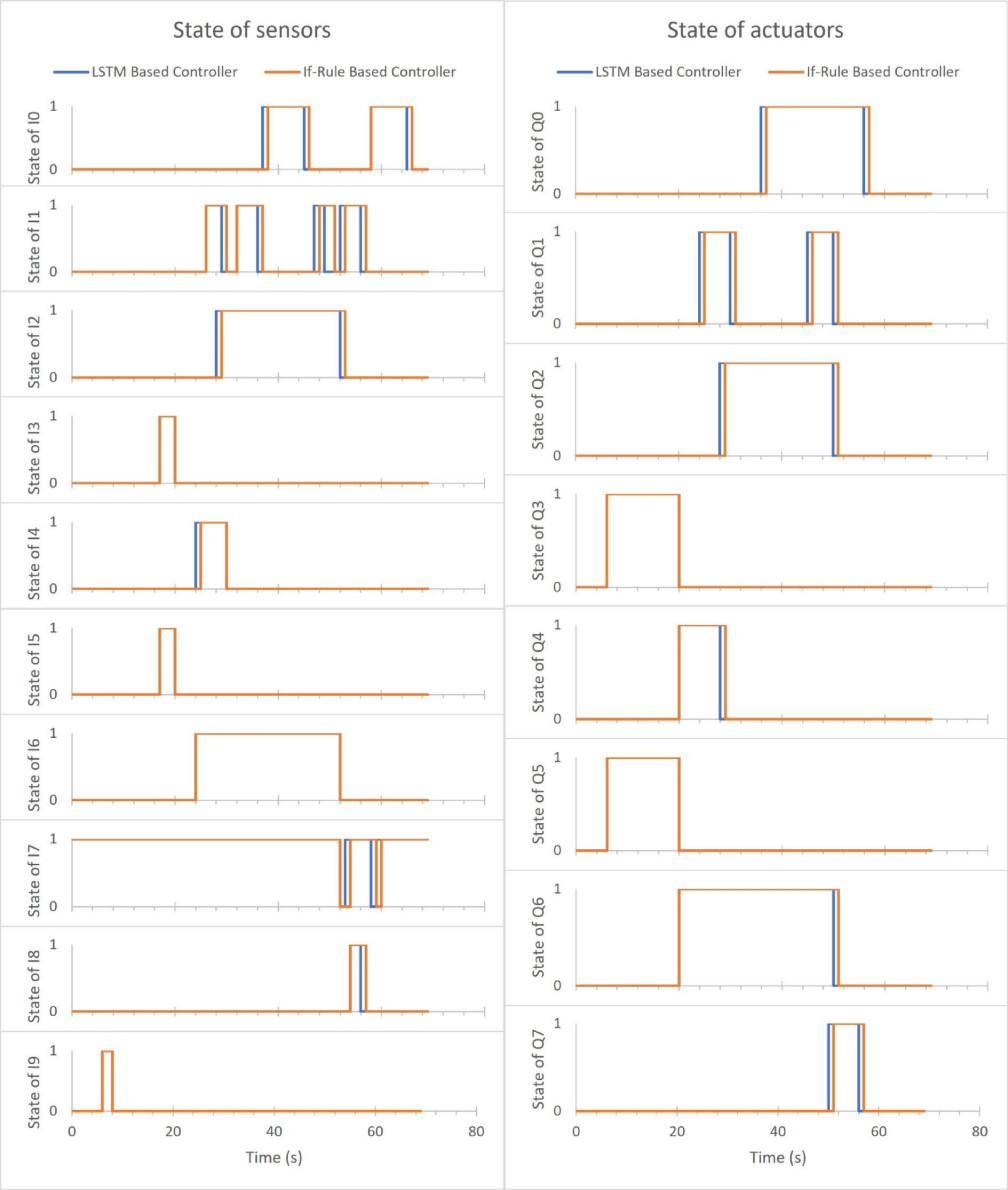
States of sensors & actuators for both controllers in the faultless scenario.

Figure 8. exhibits states of sensors & actuators for both the If-rule-based controller & LSTM-based controller in simulation one. The data was exported from the factory I/O simulator and then manipulated to depict the sensors and actuators states. The ten sensors state vs. time are demonstrated in a separate graph as shown in Fig. 8a. Each sensor has either a low or high state as it is triggered off and on. Figure 8b illustrates the eight actuators state vs. time in a separate graph. Either low or high state of each actuator as it turned off and on.

Once the start switch (I9) is pressed, the lid conveyer (Q3) and base conveyer (Q5) belts are turned on until the two proximity sensors (I3) and (I5) are changed from triggered on to off. Then the lid clamp (Q4) is turned on until the item detected is triggered on (I2), and the base clamp (Q6) is turned on until the lid reaches the base. The lid reaches the base when the signal of moving x (I0) is changed from low to high in the second time and the moving z (I2) is changed from low to high in the third time. Once the lid clamped is triggered on (I4) the move z (Q1) is turned on until the lid clamped is changed from high to low. When the item detected is triggered on (I2), the gripper is turned on until the moving z (I2) is changed from high to low in the third time. When the moving z (I1) is changed from high to low the second time, the move x (Q0) is turned on. After that, the move z (Q1) is turned on until the moving z (I1) is changed to low. Then the position rise (Q7) and base conveyor belt are turned on until the part leaving (I8) is changed from high to low. After that, the move x (Q0) and base conveyer (Q5) are turned off.

Observing and comparing the states of actuators and sensors in Fig. 8 for both if-rule-based controller and the LSTM based one, notice that the move z (Q1) delays some time before starting to work, which cause the next sequence to delay in this time, mentioning the correctly working sequence of the overall system. The accuracy of the actuators for the LSTM based controller in the faultless scenario of the case study is 92.80576%. The accuracy of the proposed approach is calculated with respect to the if-rule based controller for the time series system which is the percentage average difference of discrete data for both controllers.

Furthermore, the observation of the states of actuators & sensors in Fig. 8 serves to validate the successful operation of the assembler case study, as well as the accurate functioning of the simulation, thereby confirming the effectiveness of the proposed LSTM based controller.

**Fig. 9:**
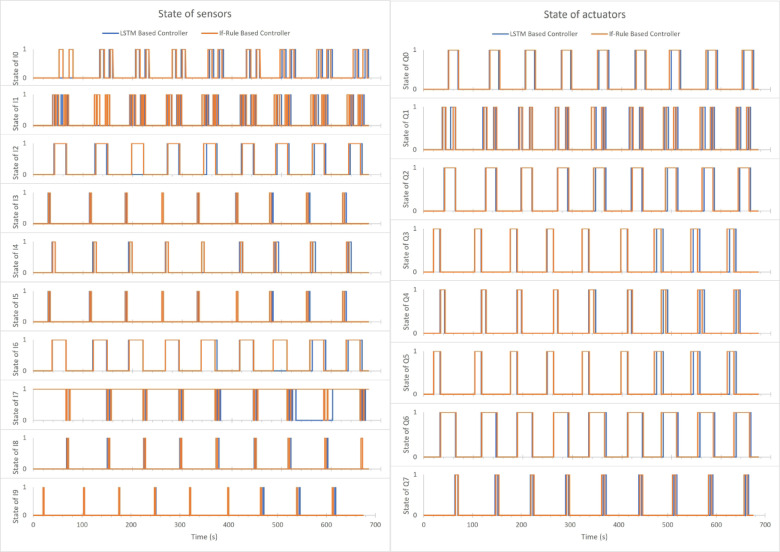
States of sensors and actuators for both controllers in Simulation 2.

Figure 9 illustrates states of sensors & actuators for both controllers in simulation 2 when creating a fault in each sensor alone. The fault started from the first sensor (I0) and then shifted in each sensor until reached the ninth sensor (I8). Therefore, the diagrams show the nine cycles of the complete overall operation process. In each cycle, one of the sensors is disabled consequently as binary codes shift. Observing and comparing the states of actuators and sensors in Fig. 9 for the if-rule-based controller and the LSTM based one, notice that:

In the first cycle, when the moving x (I0) is disabled, the second move in the z axis is worked before its default time, which affects the moving z (I1) signal.

In the fifth cycle, when the lid clamped (I4) is disabled, the move in the x axis is delayed in working, which delays move z, and grip, in addition to clamp led. Consequently, the sensors signal is delayed.

In the last three cycles when the base clamped (I6), pos. at limit (I7), and part leaving (I8) are disabled, and all actuators are delayed.

It is important to note that the nine cycles operated successfully and completed their function correctly, regardless of these delayed times.

Table 2 illustrates the accuracy of the system outputs of the LSTM based controller when creating a fault in each sensor alone in simulation two.

**Table 2:** The accuracy of the system outputs of the LSTM based controller in simulation two.

Simulation cycle	Fault sensor	Accuracy %
1	moving x (I0)	97.2067
2	moving z (I1)	93.66197
3	Item detected (I2)	96.74658
4	Lid at place (I3)	96.57534
5	Lid clamped (I4)	92.69481
6	Base at place (I5)	95.20548
7	base clamped (I6)	88.36806
8	Pos. at limit base (I7)	88.68243
9	Part leaving (I8)	89.38356

**Fig. 10:**
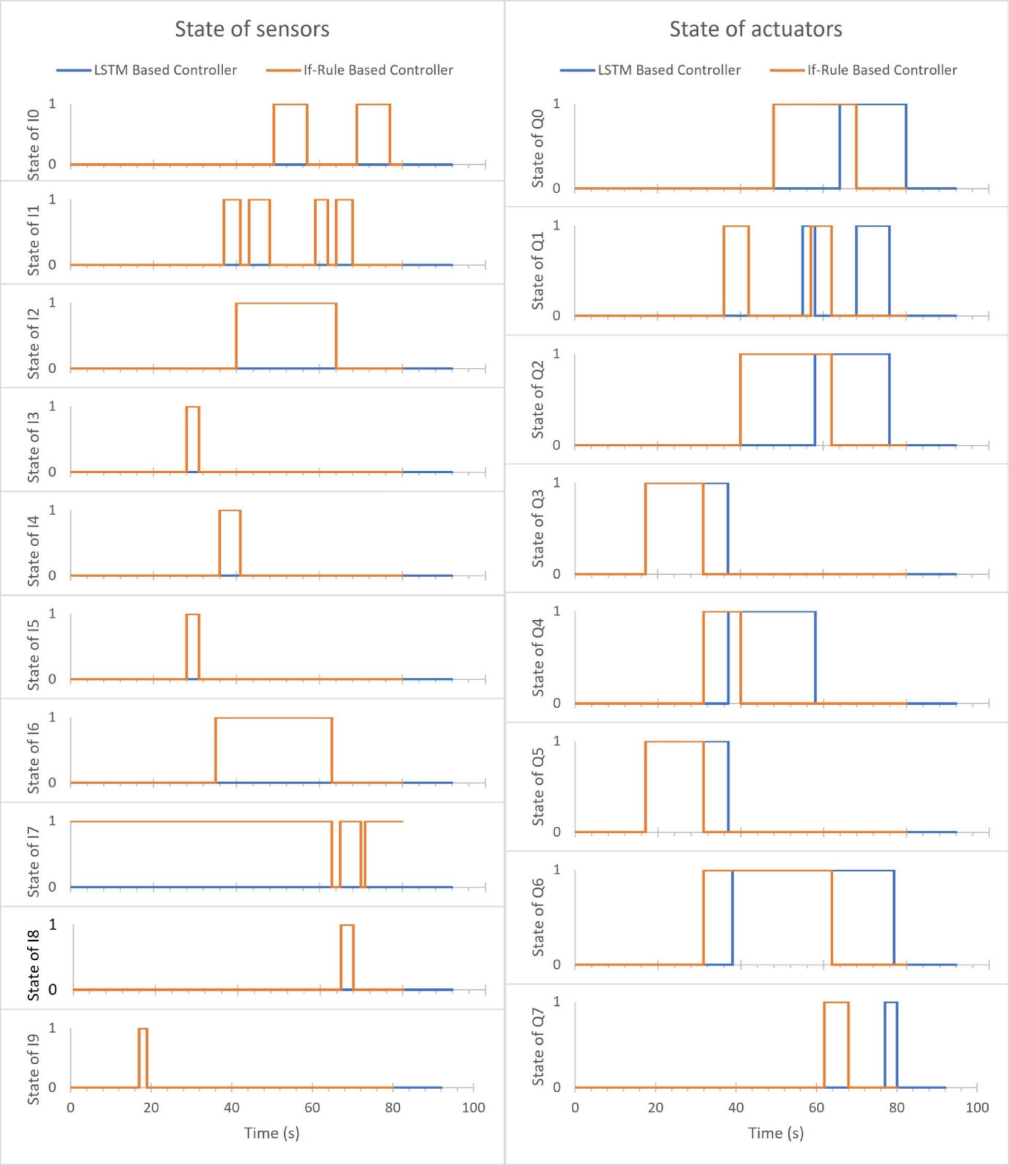
States of sensors and actuators for both controllers when the nine sensors are disabled.

The states of sensors & actuators for If-rule-based controller & LSTM based one, when the nine sensors are disabled, are presented in Fig. 10. Observing and comparing the states of actuators and sensors in Fig. 10 for the if-rule-based controller and the LSTM based controller, notice that the lid conveyer belt works more than the default time, which causes delay in the operation of move x, move z, grab, and lid clamp in addition to the base conveyer belt also work more than the default time, which causes delay in the operation of the base clamp and pos. raise. The system operated successfully and completed its function correctly regardless of these delayed times, as shown in Fig. 10 with 67.16216% accuracy.

### Discussion

LSTM can be utilized to improve FTC for DES with undiagnosable faults in sensors. LSTM has been chosen to perform as a robust controller. This controller is capable of properly controlling the AMS in the event of sensor failures. The step function is used to reform actuators’ signals. The sensors’ signals and their time sequence states are the LSTM’s inputs, whereas the LSTM’s outputs are the actuators’ signals and their time series states. Results from simulations verify the effectiveness of the proposed approach for an assembler case study in faultless cases, single faults, and multiple faults.

The acceptability of the developed FTC is an additional issue that must be addressed here. The question is whether the FTS can efficiently run the AMS in the event of any malfunction. It is crucial to acknowledge that some transitions between different states of the controlled system rely on intricate factors. The proposed LSTM-based controller can effectively maintain the appropriate control action as soon as the move from one state to the next is dependent on the time sequence. It is crucial to mention that any control system requires a minimum quantity of sensor signals to effectively carry out its control operations.

Table 3 delineates the pertinent research in FTC concerning sensor faults, categorized by application domain, methodology, kind of training datasets, sensor quantity, advantages, and limitations.

**Table 3:** Review of relevant studies in FTC concerning sensor malfunctions.

References	Methodology	Application	Advantages and limitations
^18^	PFTC utilizing a robust controller comprised of six LSTM blocks	Simulation testing of a spherical inverted pendulum	− 6 h are training time - Prolonged execution duration (85.7%) in the worst-case situation
^10^	PFTC utilizing a RNN-based robust controller	Simulation analysis of automated material transportation	- MSE = 0.00156 − 5 s are training time - 25% is the fault coverage - Suboptimal execution time of cylinder (45%) in the worst-case situation
^19^	The timed automata model with guards is the fundamental building block of AFTC.	Simulation study of automated material transportation	- Comprehensive coverage of all known faults - Elevated execution time of cylinder (14.6%) in a worst-case situation
^23^	Fault diagnosis with a hybrid autoencoder and LSTM	Simulation study of industrial process	- Enhancement in accuracy by 16.9% compared to the deep CNN.
^24^	AFTC utilizing two RNNs	Experimental testing of clamp and operation hydraulic circuit	- SSE = $\:{10}^{-4}$ − 6 + 2 min are training time
^25^	AFTC based on two LSTM	Simulation analysis of automated material transportation	- RMSE = $\:{10}^{-3}$ - Elevated fault coverage (66.6%) - 13 + 8 min are training time
Proposed approach	PFTC using an LSTM-based controller	Simulation study of an assembly production line	- RMSE = $\:{10}^{-3}$ − 17 s training time − 67.16% is the accuracy in the worst-case situation

## Conclusion

A data-driven approach is presented to develop a learning Fault-Tolerant Control (FTC) system for an Automated Manufacturing Systems (AMS) that is affected by sensor faults. The technique utilizes one single LSTM architecture. The training process reaches 6553 epochs with RMSE equivalent to $\:5\times\:{10}^{-3}$ at six minutes and 17 s. The network’s input nodes consist of the signals and time series states of the sensors, while the output nodes consist of the signals and time series states of the actuators. The applied FTC system has been eliminated the conventional diagnosis and control reconfiguration subsystems. A case study with an assembler is utilized to accomplish the recommended technique. MATLAB serves as an external controller utility that communicates with Factory I/O to demonstrate the efficacy of this case study. Results from simulations verify the effectiveness of the proposed approach that were applied on an assembler case study in faultless cases, single faults, and multiple faults. The following deductions are drawn from the positive outcomes of its utilization:

- LSTM architecture is highly beneficial for AMSs.
- The suggested system effectively fulfills its control role in both faultless scenario and different faulty scenarios.
- The faultless scenario of the assembler case study highlights the accuracy of the actuators for the LSTM-based controller.
- The accuracy of the system performance in the worst case when creating faults in the nine sensors is 67.16%.
- The accuracy of the system performance in the faultless scenario is 92.81%.
- The average accuracy of system outputs in simulation two when creating a fault in each sensor alone is 93.17%.

## Data Availability

The data used for our analyses are available in the public GitHub repository: https://github.com/MostafaHElmahdy/LSTM-based-FTC. Preprocessed outputs supporting the findings of this study are archived with a DOI at Zenodo: https://doi.org/10.5281/zenodo.15161255. Any additional datasets generated or analyzed during the current study are available from the corresponding author upon reasonable request.

## Abbreviations

AFR: Air fuel ratio
AFTC: Adaptive fault-tolerant control
AMS: Automated manufacturing systems
CNNs: Convolutional neural networks
DES: Discrete event system
FDD: Fault detection and diagnosis
FTC: Fault-tolerant control
FTSS: Fast terminal sliding mode surface
IC: Internal combustion
LSTM: Long short-term memory
MJSs: Markovian jump systems
NN: Neural network
PFTCS: Passive fault tolerant control system
PI: Proportional plus Integral
RBF: Radial basis function
RMSE: Root mean square error
RNNs: Recurrent neural networks
SSE: Sum of squared errors
TSMC: Terminal sliding mode control

## List of symbols

b: Bias
t: Time step
w: Weight
x: Input state
R: Recurrent weight
h: Hidden state
c: Cell state

## Greek letters

${\beta }_{v}$: Squared decay factor
∇: Gradient
E(θ): Loss function
ɛ: Epsilon
m: Parameter gradient
α: Learning rate
$${\beta }_{m}$$: Decay factor
θ: Parameter vector
υ: Squared parameter gradient

## Subscripts

ƒ: Forget gate
g: Layer input
i: Input gate
o: Output gate

## References

[1] Leng, J. et al. ManuChain II: blockchained smart contract system as the digital twin of decentralized autonomous manufacturing toward resilience in industry 5.0. *IEEE Trans. Syst. Man. Cybern Syst.* **53**(8), 4715–4728. 10.1109/TSMC.2023.3257172 (2023).

[2] Morgan, J., Halton, M., Qiao, Y. & Breslin, J. G. Industry 4.0 smart reconfigurable manufacturing machines. *J. Manuf. Syst.* **59**(April), 481–506. 10.1016/j.jmsy.2021.03.001 (2021).

[3] Koren, I. & Krishna, C. M. *Fault-tolerant Systems* (Morgan Kaufmann, 2020).

[4] Nguyen, V. C., Vo, A. T. & Kang, H. J. A finite-time fault-tolerant control using non-singular fast terminal sliding mode control and third-order sliding mode observer for robotic manipulators. *IEEE Access* **9**, 31225–31235. 10.1109/ACCESS.2021.3059897 (2021).

[5] Abbadi, R. E. & Jamouli, H. Active fault-tolerant control of networked systems with packet losses. *Int. J. Dyn. Control* **12**(7), 2437–2446. 10.1007/s40435-023-01352-w (2024).

[6] Wang, Z. Facility Control and Optimization Problems in Data Centers. 10.1007/978-3-030-44184-5_100079 (2021).

[7] Abbaspour, A., Mokhtari, S., Sargolzaei, A. & Yen, K. K. A survey on active fault-tolerant control systems. *Electron* **9**(9), 1–23. 10.3390/electronics9091513 (2020).

[8] Brito, L. C., Susto, G. A., Brito, J. N. & Duarte, M. A. V. An explainable artificial intelligence approach for unsupervised fault detection and diagnosis in rotating machinery. *Mech. Syst. Signal. Process.* **163**, 108105. 10.1016/j.ymssp.2021.108105 (2022).

[9] Zhang, Q. Dynamic system fault diagnosis under sparseness assumption. *IEEE Trans. Signal. Process.* **69**, 2499–2508. 10.1109/TSP.2021.3072004 (2021).

[10] El-Mahdy, M. H., Maged, S. A. & Awad, M. I. End-to-End Fault Tolerant Control of Discrete Event System Using Recurrent Neural Networks, *MIUCC –2nd Int. Mobile, Intelligent, Ubiquitous Comput. Conf.*, 266–271 (2022). 10.1109/MIUCC55081.2022.9781748.

[11] Ali, K., Mehmood, A. & Iqbal, J. Fault-tolerant scheme for robotic manipulator—nonlinear robust back-stepping control with friction compensation, *PLoS One* **16**(8), e0256491. 10.1371/journal.pone.0256491 (2021). 10.1371/journal.pone.0256491 PMC8378759 34415970

[12] Scholar, A. & Wardell, D. C. Deep Learning-based, passive fault tolerant control facilitated by a taxonomy of cyber-attack effects (2020).

[13] Vo, A. T. & Kang, H. J. Fault-Tolerant control method for robot manipulators based on non-singular fast terminal sliding mode control and disturbance observer. *IEEE Access.* **8**, 109388–109400. 10.1109/ACCESS.2020.3001391 (2020).

[14] Le, Q. D. & Kang, H. J. Finite-time fault-tolerant control for a robot manipulator based on synchronous terminal sliding mode control. *Appl. Sci.* **10**(9). 10.3390/app10092998 (2020).

[15] M. Van and D. Ceglarek, Robust fault tolerant control of robot manipulators with global fixed-time convergence. *J. Frankl. Inst.* **358**(1), 699–722. 10.1016/j.jfranklin.2020.11.002 (2021).

[16] Amin, A. A. & Mahmood-ul-Hasan, K. Robust passive fault tolerant control for air fuel ratio control of internal combustion gasoline engine for sensor and actuator faults. *IETE J. Res.* **69**(5), 2846–2861. 10.1080/03772063.2021.1906767 (2023).

[17] Yang, H., Yin, S. & Kaynak, O. Neural network-based adaptive fault-tolerant control for Markovian jump systems with nonlinearity and actuator faults. *IEEE Trans. Syst. Man. Cybern Syst.* **51**(6), 3687–3698. 10.1109/TSMC.2020.3004659 (2021).

[18] Baimukashev, D., Rakhim, B., Rubagotti, M. & Varol, H. A. End-to-end deep fault-tolerant control. *IEEE/ASME Trans. Mech.* **27**(4), 2224–2234. 10.1109/TMECH.2021.3100150 (2022).

[19] El-Mahdy, M. H., Maged, S. A. & Awad, M. I. Active Fault Tolerant Control of Discrete Event System Subjected to Sensors Fault. in *17th Int. Comput. Eng. Conf. ICENCO 2021* 24–29 (2021). 10.1109/ICENCO49852.2021.9698832.

[20] Hagh, Y. S., Asl, R. M., Fekih, A., Wu, H. & Handroos, H. Active fault-tolerant control design for actuator fault mitigation in robotic manipulators. *IEEE Access* **9**, 47912–47929. 10.1109/ACCESS.2021.3068448 (2021).

[21] Chang, X., Rong, L., Chen, K. & Fu, W. LSTM-based output-constrained adaptive fault-tolerant control for fixed-wing UAV with high dynamic disturbances and actuator faults. *Math. Probl. Eng.* **2021**. 10.1155/2021/8882312 (2021).

[22] El-Mahdy, M. H., Awad, M. I. & Maged, S. A. Deep-learning-based design of active fault-tolerant control for automated manufacturing systems subjected to faulty sensors. *Trans. Inst. Meas. Control*10.1177/01423312241229493 (2024).

[23] Park, P., Di Marco, P., Shin, H. & Bang, J. Fault detection and diagnosis using combined autoencoder and long short-term memory network. *Sensors (Switzerland)*. **19**(21), 1–17. 10.3390/s19214612 (2019). 10.3390/s19214612 PMC6866134 31652821

[24] Abdelhameed, M. M. & Darabi, H. Neural network based design of fault-tolerant controllers for automated sequential manufacturing systems. *Mechatronics* **19**(5), 705–714. 10.1016/j.mechatronics.2009.02.007 (2009).

[25] El-Mahdy, M. H., Awad, M. I. & Maged, S. A. Deep-learning-based design of active fault-tolerant control for automated manufacturing systems subjected to faulty sensors, *Trans. Inst. Meas. Control* **46**(12), 2289–2299. 10.1177/01423312241229493 (2024).

[26] Hochreiter, S. & Schmidhuber, J. Long short-term memory. *Neural Comput.* **9**(8), 1735–1780. 10.1162/NECO.1997.9.8.1735 (1997). 10.1162/neco.1997.9.8.1735 9377276

[27] Sadrossadat, M. M. A. S. A. & Derhami, V. Long short-term memory neural networks for modeling nonlinear electronic components. *IEEE Trans. Compon. Packag Manuf. Technol.* **11**(5), 840–847. 10.1109/TCPMT.2021.3071351 (2021).

[28] Kang, H., Yang, S., Huang, J. & Oh, J. Time series prediction of wastewater flow rate by bidirectional LSTM deep learning. *Int. J. Control Autom. Syst.* **18**(12), 3023–3030. 10.1007/s12555-019-0984-6 (2020).

[29] Battesini, M., ten Caten, C. S. & de Pacheco, D. A. Key factors for operational performance in manufacturing systems: conceptual model, systematic literature review and implications. *J. Manuf. Syst.* **60**, 265–282. 10.1016/j.jmsy.2021.06.005 (2021).

[30] Pandey, S. K., Mishra, R. B. & Tripathi, A. K. Machine learning based methods for software fault prediction: A survey. *Expert Syst. Appl.* **172**, 114595. 10.1016/j.eswa.2021.114595 (2021).

[31] Reghenzani, F., Guo, Z. & Fornaciari, W. Software fault tolerance in Real-Time systems: identifying the future research questions. *ACM Comput. Surv.* **55**(14). 10.1145/3589950 (2023).

[32] Kingma, D. P. & Ba, J. L. Adam: A method for stochastic optimization. in *3rd Int. Conf. Learn. Represent. ICLR 2015 - Conf. Track Proc.* 1–15 (2015).

[33] Mehmood, F., Ahmad, S. & Whangbo, T. K. An efficient optimization technique for training deep neural networks. *Mathematics* **11**(6). 10.3390/math11061360 (2023).

[34] Reyad, M., Sarhan, A. M. & Arafa, M. A modified Adam algorithm for deep neural network optimization. *Neural Comput. Appl.* **35**(23), 17095–17112. 10.1007/s00521-023-08568-z (2023).

[35] El-Mahdy, M. H., Awad, M. I. & Maged, S. A. Deep-learning-based design of active fault-tolerant control for automated manufacturing systems subjected to faulty sensors. *Trans. Inst. Meas. Control*. **46**(12), 2289–2299. 10.1177/01423312241229493 (2024).

[36] Amin, A. A., Sajid Iqbal, M. & Hamza Shahbaz, M. Development of intelligent fault-tolerant control systems with machine learning, deep learning, and transfer learning algorithms: A review. *Expert Syst. Appl.* **238**, 121956. 10.1016/j.eswa.2023.121956 (2024).

[37] Sotskov, Y. N. Assembly and production line designing, balancing and scheduling with inaccurate data: A survey and perspectives. *Algorithms* **16**(2). 10.3390/a16020100 (2023).

[38] Kritzinger, W., Karner, M., Traar, G., Henjes, J. & Sihn, W. Digital twin in manufacturing: A categorical literature review and classification. *IFAC-PapersOnLine* **51**(11), 1016–1022. 10.1016/j.ifacol.2018.08.474 (2018).

